# Serological identification and expression analysis of gastric cancer-associated genes

**DOI:** 10.1038/sj.bjc.6600321

**Published:** 2002-06-05

**Authors:** A Linē, A Stengrēvics, Z Slucka, G Li, E Jankevics, R C Rees

**Affiliations:** Biomedical Research and Study Centre, University of Latvia, 1 Ratsupites St, LV-1067, Riga, Latvia; Latvian Oncology Center, 4 Hipokrata St, LV-1079, Riga, Latvia; Department of Life Sciences, Nottingham Trent University, Clifton Lane, Nottingham NG11 8NS, UK

**Keywords:** SEREX, tumour antigens, autoantibodies, TACC1, Tbdn-1, NUCB2

## Abstract

Serological identification of tumour antigens by recombinant expression cloning has proved to be an effective strategy for the identification of cancer-associated genes having a relevance to cancer aetiology and progression, and for defining possible targets for immunotherapeutic intervention. In the present study we applied this technique to identify immunogenic proteins for gastric cancer that resulted in isolation of 14 distinct serum-reactive antigens. In order to evaluate their role in tumourigenesis and assess the immunogenicity of the identified antigens, we characterised each cDNA clone by DNA sequence analysis, mRNA tissue distribution, comparison of mRNA levels in cancerous and adjacent non-cancerous tissues and the frequency of antibody responses in allogeneic patient and control sera. Previously unknown splice variants of TACC1 and an uncharacterised gene Ga50 were identified. The expression of a newly identified TACC1 isoform is restricted to brain and gastric cancer tissues. Comparison of mRNA levels by semi-quantitative RT–PCR revealed a relative overexpression of three genes in cancer tissues, including growth factor granulin and Tbdn-1 – an orthologue of the mouse acetyltransferase gene which is associated with blood vessel development. An unusual DNA polymorphism – a three-nucleotide deletion was found in NUCB2 cDNA but its mRNA level was consistently decreased in gastric tumours compared with that in the adjacent non-cancerous tissues. This study has revealed several new gastric cancer candidate genes; additional studies are required to gain a deeper insight into their role in the tumorigenesis and their potential as therapeutic targets.

*British Journal of Cancer* (2002) **86**, 1824–1830. doi:10.1038/sj.bjc.6600321 www.bjcancer.com

© 2002 Cancer Research UK

## 

Cancer development is a multistep process, during which the cell acquires new phenotypic traits (overriding growth controls, induction of angiogenesis, evasion from host anti-tumour responses, extravasation and growth at metastatic sites etc.) as a result of successive genetic alterations (Hanahan and Weinberg, 2000). Identification of genetic/epigenetic alterations that contribute to the malignant phenotype capabilities is of major importance for the understanding the molecular basis of cancer, defining possible targets for therapeutic intervention, prognosis and diagnosis of human malignancies. Sahin *et al* (1995) introduced a novel approach – serological identification of tumour antigens by recombinant expression cloning – called SEREX, for the identification of tumour antigens recognised by cancer patient auto-antibodies. This strategy is based on the construction of cDNA expression libraries from tumour specimens and immunoscreening of the libraries with cancer patient sera, thus allowing a systematic search for immunoreactive proteins (Sahin *et al*, 1995). SEREX has been applied to multiple human tumours, including melanoma (Jager *et al*, 2000a), renal cell cancer (Scanlan *et al*, 1999), astrocytoma (Sahin *et al*, 1995), breast (Jager *et al*, 1999) and colon (Scanlan *et al*, 1998), identifying tumour antigens for each tumour type, indicating that a humoral response is elicited in the majority of cancer patients.

SEREX-defined antigens represent a broad spectrum of structurally and functionally diverse proteins – transcription factors, adhesion molecules, signalling molecules, metabolic enzymes and cDNA sequences of 1549 antigens have been deposited in the SEREX database. Several categories of antigens have been detected by SEREX i.e. shared cancer-testis (C-T) antigens, differentiation antigens, mutated genes and translocation products, splice variant products, overexpressed antigens, viral antigens, cancer-related and cancer-independent autoantigens (Tureci *et al*, 1997; Sahin *et al*, 2001). Although the majority of the SEREX-identified genes have not been characterised beyond the preliminary sequence analysis, several of them have been proposed as attractive candidates for the construction of anti-cancer vaccines. For instance, ‘cancer-testis’ antigen NY-ESO-1 was identified by SEREX in oesophageal cancer and is regarded as one of the most immunogenic tumour antigens; antibody responses to NY-ESO-1 have been observed in 40–50% of patients with NY-ESO-1 expressing tumours and antibody production strongly correlates with CD8+ T cell responses in these patients (Jager *et al*, 2000c). Clinical trials, investigating the immunological effects of vaccination with NY-ESO-1 peptides, are ongoing (Jager *et al*, 2000b). Several previously known cytotoxic T cell targets, for example tyrosinase and MAGE antigens, have been detected by SEREX, supporting the premise of integrated CTL and B cell responses to tumour antigens. Moreover, antibody responses to mutated p53 (Scanlan *et al*, 1998), putative tumour suppressor ING1 (Jager *et al*, 1999) and amplified translation factor eIF-γ4 (Brass *et al*, 1997) have been detected by SEREX, thus demonstrating the potential of this technique for identification of genes that play a role in cancer aetiology and may serve as diagnostic markers or indicators of progression of the disease.

In the present study we applied SEREX to identify clinically relevant cancer-associated genes in human gastric carcinoma, and to define further the spectrum of immunogenic proteins in cancer. Fourteen different antigens were recognised by cancer patients' sera and were further characterised by sequence analysis, mRNA expression pattern and reactivity with allogeneic sera.

## MATERIALS AND METHODS

### Tissue specimens and patient sera

Gastric cancer and the adjacent non-cancerous tissue specimens were resected and snap frozen immediately after surgery. Tissue specimens and sera were obtained from 20 gastric cancer patients who had undergone surgical resection at the Latvian Oncology Center after the informed written consent was obtained (the study has been approved by the local ethical review board). In addition, serum samples were obtained from stomach, colon, breast and prostate cancer patients undergoing diagnostic procedures and from healthy volunteers.

### Construction of cDNA expression library

A cDNA expression library was constructed from a tumour specimen of a moderately differentiated, ulcerated gastric adenocarcinoma. Total RNA was isolated using Trizol reagent according to manufacturer's protocol (Life Technologies, Inc.). Poly(A)^+^ RNA was purified from total RNA using Dynabeads mRNA Purification kit (Dynal AS, Norway) and cDNA was ligated into the lambda Uni-ZAP XR vector using Gigapack III Gold cloning kit (Stratagene GmbH). After *in vitro* packaging, library containing 2×10^6^ primary cDNA clones was obtained.

### Immunoscreening

Immunoscreening of cDNA library was performed as described by Sahin *et al* (1995). Briefly, after one round of amplification, the cDNA library was screened with 1 : 250 diluted autologous patient's serum and allogeneic sera, which had been previously preabsorbed with *E coli*-phage lysate. In order to eliminate cDNA clones encoding human IgG, nitrocellulose membranes containing phage plaques were pre-screened with AP-conjugated rabbit anti-human IgG secondary antibody (Pierce, USA) and reactive plaques were marked to exclude them from further study. Membranes were then incubated with patients' sera, and serum-reactive clones were detected with AP-conjugated secondary antibody and visualised by incubating with 5-bromo-4chloro-3-indolyl-phosphate and nitroblue tetrazolium. The reactive phage clones were subcloned to monoclonality and converted to pBluescript phagemids.

### DNA sequencing and sequence analysis

Plasmid DNA was purified using QIAprep Spin Miniprep kit (QIAGEN GmbH), analysed by *Eco*RI*/Xho*I restriction enzyme digestion and clones representing different cDNA inserts were sequenced using BigDye Terminator Cycle Sequencing Ready Reaction kit on ABI PRISM 310 automatic sequencer (Applied Biosystems). Gene-specific primers were designed to obtain full insert sequences. cDNAs were identified by homology search through GenBank (www.ncbi.nlm.nih.gov). Multiple sequence alignments were performed with DNASIS (Hitachi Software Engineering Co Ltd) and MACAW (NCBI) software. Chromosomal localisation and exon–intron organisation for uncharacterised cDNAs was determined by comparison to the working draft of the human genome. Putative protein domains were predicted by scanning the sequences against PROSITE and Pfam databases (www.expasy.org and www-ludwig.unil.ch/SEREX).

### Detection of antibodies in allogeneic sera

To assess frequencies of antibody responses to the SEREX-defined antigens in allogeneic sera, slightly modified immunoscreening procedure was used. *E coli* were transfected directly on gridded agar plate, by spotting 1 μl of monoclonal positive phage (20–30 pfu μl^−1^) side by side with non-recombinant phages. ‘Phage arrays’ were screened with 1 : 200 diluted allogeneic sera as described above, excluding the IgG pre-screening step.

### Comparative RT–PCR analysis

The mRNA expression pattern of SEREX-defined antigens was analysed by RT–PCR in a panel of normal tissue RNA from whole brain, liver, heart, kidney, lung, trachea (Clontech), spleen, colon, stomach, testes, ovary (Ambion), PBLs and a specimen of gastric cancer and adjacent tissues. Relative mRNA levels were compared between cancerous and adjacent non-cancerous tissues by semi-quantitative RT–PCR. Total RNA was isolated from paired tissue specimens using Trizol reagent according to manufacturer's protocol (Life Technologies, Inc.). The first-strand cDNA was synthesised from 4 μg of total RNA primed with oligo-dT(18) and random hexamer primers using First-Strand cDNA Synthesis Kit (Fermentas, Lithuania). Gene specific PCR primers located within different exons were designed to amplify cDNA fragments (300–400 bp in length) of nine SEREX-defined genes and GAPDH, β-actin and histone H4 were used as internal standard genes. One fiftieth of RT mixture was amplified in GeneAmp PCR System 2400 thermal cycler (Perkin-Elmer Corp.) in a total reaction volume of 20 μl containing 10 pmole of each primer, 200 μM of dNTPs and 2 U of Taq polymerase (Fermentas, Lithuania). Optimisation of cycling conditions (amount of input cDNA and number of cycles) was performed as described by Toh *et al* (1997). Amplification of all target genes was performed simultaneously, at the same cycling conditions (1 min at 94°C, 30 s at 58°C, 45 s at 72°C), except for the number of cycles that was determined for each target gene (20 for β-actin, 25 for GAPDH, 27 for Zg4, 28 for Ga55 and Ga19, 29 for Ga27, Ga50, Zg2, histone H4 and 30 for Ga34, Zg14 and Zg15). Quantitative analysis of RT–PCR products was performed densitometrically after scanning the ethidium bromide stained gel on digital gel documentation and analysis system GDS8000 (Ultra-Violet Products Ltd, UK) and the intensities of bands were calculated using GelWorks software. Standard curves of amplification of each target gene were constructed from series of PCRs with ten 1.5-fold dilutions of normal stomach cDNA. Amounts of PCR products were linearly dependent on input cDNA over 10-fold dilutions of cDNA. The relative amounts of each mRNA were normalised to GAPDH and β-actin. The obtained values in tumours (T) were compared to those in matched normal epithelium (N) and T/N ratios were calculated for each mRNA from each patient's tissue samples. Each reaction was performed in duplicate. Ratios ⩾2 or ⩽0.5 (the mean values of two independent experiments) were considered to represent significant alteration of expression levels.

### 5′ and 3′ RACE analysis

5′ and 3′ ends of full-length cDNAs were cloned from gastric cancer and adjacent tissues using FirstChoise™ RLM-RACE kit (Ambion) according to manufacturer's protocol. Briefly, for cloning of 5′-ends, 10 μg of total RNA was treated with Calf Intestinal Phosphatase to remove 5′-phosphates from un-capped RNAs, the cap structure was removed from the full-length mRNA by Tobacco Acid Pyrophosphatase and RNA adapters were ligated to mRNA molecules containing 5′-phosphate. A random-primed reverse transcription and nested PCR with gene-specific and adapter-specific primers were performed and products were cloned using InsT/Aclone™ PCR Product Cloning Kit (Fermentas, Lithuania). For cloning of 3′ends, 1 μg of total RNA was reverse transcribed using an oligo (dT)-anchored 3′RACE adapter and nested PCR was performed.

## RESULTS

### Identification of serum-reactive cDNA clones

Eleven serum-reactive clones were identified by immunoscreening of approximately 8×10^5^ clones from gastric cancer cDNA expression library with autologous patient's serum. In addition ∼3×10^5^ phage plaques were screened using allogeneic serum from a patient with poorly differentiated infiltrative gastric adenocarcinoma, resulting in the isolation of nine positive clones. Full insert sequences of serum-reactive cDNA clones were obtained and identified by homology search through GenBank and SEREX databases. This revealed that serum-reactive clones are derived from 14 distinct genes, including eight genes whose function is unknown (listed in Table 1

**Table 1:**
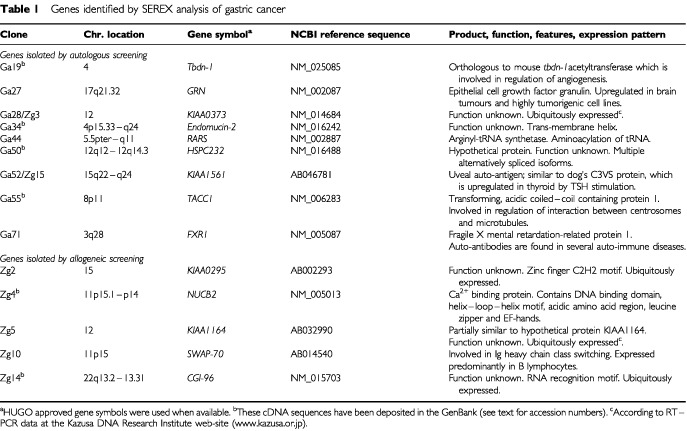
Genes identified by SEREX analysis of gastric cancer

). Only two genes were detected by both autologous and allogeneic screening.

The frequency of antibodies from cancer patients and controls reacting with SEREX antigens was determined. Seven of the antigens had similar reactivity with serum from cancer patients and control individuals, three antigens reacted exclusively with autologous patient serum, while four antigens reacted with several cancer patient sera but not with sera from healthy individuals (Table 2

**Table 2:**
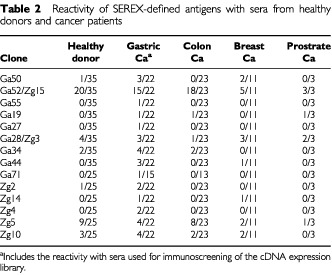
Reactivity of SEREX-defined antigens with sera from healthy donors and cancer patients

).

### Analysis of sequence variations

Several sequence variations were found in three cancer-derived cDNAs in comparison with GenBank entries. An alteration of codon GAC (nt394) to GGC (Asp→Gly) in clone Ga34 (deposited in GenBank, AY039241) encoding Endomucin2 (NM_016242) and three scattered nucleotide insertions within the 136 bp region of Zg14 (deposited in GenBank, AY039240) encoding for CGI-96 (AF151854), resulting in an altered 45 amino acid region in the predicted protein sequence were also observed. However sequencing of RT–PCR products derived from autologous normal gastric epithelium and tissue samples of two other patients revealed the same sequence variant for both genes, thus ruling out the possibility of somatic mutation in cancer cells.

Direct sequencing of NUCB2 cDNA derived from cancerous tissue of the patient whose serum was used for allogeneic screening showed heterogeneity of RT–PCR products at the 3′ end of the coding region which is not present in the clone Zg4. The RT–PCR product was cloned into pTZ57R/T vector and two of five clones sequenced had ACA deletion (submitted to GenBank, AF450266). The 3-nucleotide deletion was also demonstrated by sequencing of amplified genomic DNA fragment. As the normal tissue sample from this patient was not available we could not directly determine whether this deletion represent somatic mutation or allelic polymorphism. Next we analysed DNA sequences from tumour and adjacent tissue samples of five gastric cancer patients, tumour and blood samples of five breast cancer patients and from blood samples of 25 donors. All paired tissue specimens had the same genotype thus showing that the deletion represents a germline DNA polymorphism and likely is not an immunogenic stimuli for production of anti-NUCB2 antibodies in the patients. Nevertheless, all gastric cancer patients and two breast cancer patients were heterozygous, one breast cancer patient had ACA deletion on both alleles, however five of 25 donors were also heterozygous at this locus, thus showing very high frequency of this unusual polymorphism not only among cancer patients but also in a general population.

### Identification of TACC1 and HSPC232 splice variants

**Figure 1:**
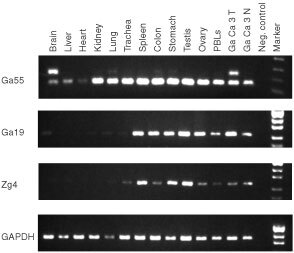
Tissue distribution of Ga55 (*TACC1*), Ga19 (*Tbdn-1*), Zg4 (*NUCB2*) analysed by RT–PCR. Primers used were: Ga55 forward 5′-ACCCAAGAAGGCAAAGTCGC-3′ and Ga55 reverse 5′-AAATCTGGGAGATCACTGCC-3′. The primers are flanking the alternative exon – demonstrating the presence of the two splice variants, 119 and 155 bp. Ga19 forward primer 5′-CTAAAATCTATAAGCATGCTGG-3′, Ga19 reverse 5′-ATTCATTGCTTTATAAGCCTGG-3′, Zg4 forward 5′-TCAAGCAAGTGATTGATGTGC-3′, Zg4 reverse 5′-TCAGGATTCAGGTGGTTTAGG-3′, Fragment of GAPDH cDNA was amplified as an internal control. 32, 29, 27, and 25 cycles of amplification were used for Ga55, Ga19, Zg4 and GAPDH, respectively. Ga Ca 3T and Ga Ca 3N – tumour and adjacent tissues from gastric cancer patient.

Comparison of Ga55 cDNA (submitted to GenBank, AY039239) with the published TACC1 sequence (GenBank, AF049910) showed that Ga55 represents a TACC1 isoform generated by inclusion of alternative 36 bp exon. The alternative exon is present in another GenBank sequence (KIAA1103, AB029026) derived from brain tissue, but is skipped in all other EST sequences, indicating that TACC1 pre-mRNA splicing may be regulated in tissue specific manner. The mRNA expression pattern of TACC1 isoforms was analysed by RT–PCR in a panel of RNAs derived from normal brain, liver, heart, kidney, lung, trachea, spleen, colon, stomach, testis, ovary, PBLs and in gastric cancer and adjacent non-cancerous tissue specimens using primers flanking the alternative exon. The transcript variant lacking the alternative exon was detected universally in all tissues tested. In contrast, the transcript containing the 36 bp exon was strongly expressed in brain and gastric cancer tissues; just trace amounts were detectable in lung and colon, but not in normal stomach and other normal tissues analysed (Figure 1

). In subsequent analysis of paired gastric cancer and normal tissues, inclusion of the alternative exon was detected in three of 20 cancer tissue specimens but not in the adjacent tissues.

Clone Ga50 represents a novel splice variant of uncharacterised gene HSPC232. Alignment of GenBank cDNA entries and corresponding ESTs revealed at least four transcript variants differing in their 5′ and 3′ regions. RLM–RACE analysis showed that all splice variants are expressed in gastric cancer and adjacent tissues, and no additional variants were found. RT–PCR analysis showed that the splice variants are universally expressed in normal tissues but the efficiency of alternative splicing seems to be differentially regulated in different tissues. However no significant differences in expression of the splice variants in gastric cancer and adjacent tissues were found.

### mRNA expression pattern of SEREX-identified antigens

Multiple matching EST sequences were found in GenBank/EMBL databases for all of the identified antigens, however some genes shared sequence identity only to ESTs derived from cancerous tissues. Therefore, we analysed the mRNA tissue distribution of selected antigens by RT–PCR in normal tissues (brain, liver, heart, trachea, lung, kidney, spleen, colon, stomach, testis, ovary and PBLs) and in gastric cancer and adjacent epithelium. Ga19, Zg15 and Zg4 mRNAs showed differential tissue distribution pattern. Relatively high expression of Ga19 and Zg15 was observed in normal testis, ovary, spleen, stomach, colon, and gastric cancer while it was weak or undetectable in other tissues tested. Zg4 was predominantly expressed in spleen, testis and normal stomach whereas RT–PCR signals were faint in all other tissues including cancerous and adjacent non-cancerous tissues from a gastric cancer patient (Figure 1).

### Comparison of mRNA levels in cancerous and adjacent normal tissues

**Figure 2:**
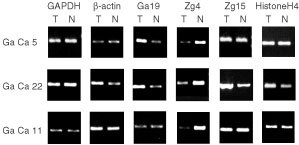
Comparison of Ga19, Zg4 and Zg15 mRNA levels in three paired gastric cancer (T) and adjacent epithelium (N) samples by semi-quantitative RT–PCR. The same primers as shown in Figure 1 were used for amplification of Ga19 and Zg4. Primers used for Zg15 were: forward primer 5′-TCTGGAACTATCTTTACCCAG-3′, reverse 5′-GTTTGACGTGCTGAGCAAGC-3′. GAPDH and β-actin are used as internal standards. Histone H4 is used as a marker for the rate of cell proliferation.

In order to evaluate whether expression levels of genes encoding SEREX-antigens are altered in cancer tissues, we compared the relative mRNA levels in cancerous and paired non-cancerous tissues by semi-quantitative RT–PCR. Expression levels of nine genes isolated from gastric cancer cDNA library (Ga19, Ga27, Ga34, Ga50, Ga55, Zg2, Zg4, Zg14, and Zg15) were analysed in paired tissue samples from 20 gastric cancer patients. Relative mRNA levels of target genes were normalised to GAPDH and β-actin, and Tumour/Normal (T/N) ratios were calculated. Each T/N value represents the mean value of two independent experiments usually differing in less than 20%. T/N values 2 or ⩽0.5 were considered to represent significant difference. Histone H4 cDNA was amplified as a marker for the rate of proliferating cells in the tissue sample. Relative overexpression of three genes in various numbers of tumour specimens was observed. An example of the RT–PCR results is shown in Figure 2

. We observed 2.0–6.5-fold increase in mRNA level of Ga19 in three of 20, 2.3–7.4-fold increase of Zg15 in three of 20 and 2.3- and 2.9-fold increase of Ga27 in two of nine gastric cancer specimens in comparison with matched normal tissues (when expression levels were normalised to β-actin). In the remaining cases, mRNA levels of these genes were similar in cancer and non-cancerous tissues. Interestingly, 2–11-fold downregulation of Zg4 was observed in 10 of 20 gastric cancer specimens. The major drawback for measurement of the relative abundance of cDNAs in clinical samples is the lack of an invariable internal standard. Expression levels of commonly used standards such as actins, tubulins and GAPDH may vary among different cell types (Weisinger *et al*, 1999) and in response to various factors such as hypoxia and, surgical resection (Thellin *et al*, 1999; Zhong and Simons, 1999) etc. For example, we observed apparently increased GAPDH mRNA levels in two tumour samples relative to β-actin and to target genes, resulting in down-estimation of the real expression levels of target genes when normalised to GAPDH.

### Sequence analysis of Ga19 cDNA

Sequence analysis of the Ga19 clone showed an uninterrupted ORF throughout the insert sequence indicating that the cDNA represented by this clone is truncated at both 5′ and 3′-ends. To obtain the complete cDNA sequence RLM-RACE was performed. A single fragment of 930 bp was obtained in 5′RLM–RACE analysis and no variation of the transcription start site was found, but 3′RACE yielded in an 850 bp product. The assembled cDNA sequence contains 281 bp 5′UTR with several stop codons, 2595 bp coding sequence and 206 bp 3′UTR (deposited in GenBank, AY039242). Scanning of the predicted amino acid sequence against PROSITE and Pfam databases revealed N-terminal acetyltransferase domain (amino acids 250–602), bipartite nuclear localisation signal (aa 612–629) and TPR repeat region (aa 80–113; 46–181; 374–441).

## DISCUSSION

Definition of all immunogenic proteins in cancer (‘cancer immunome’) is regarded as the main long-term goal of SEREX (Jager *et al*, 1999; Pfreundschuh, 2000). Four of 14 antigens identified in the current study have been previously detected by SEREX in various tumour types (Table 3

**Table 3:**
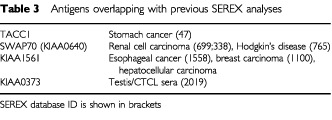
Antigens overlapping with previous SEREX analyses

), and two of the identified genes – epithelial cell growth factor granulin (Lu and Serrero, 2000; Liau *et al*, 2000) and transforming coiled coil containing gene TACC1 (Still *et al*, 1999) have been previously shown to be associated with cancer. A further gene – FXR1 is autoantigen known to elicit antibody production in scleroderma patients (Bolivar *et al*, 1998). Another isolated gene is an Agr-tRNA synthetase. Autoantibodies against particular aminoacyl-tRNA synthetases are frequently found in myositis, interstitial lung disease, and artritis (Hirakata *et al*, 1999) and various tRNA synthetases also have been detected by SEREX in renal (Scanlan *et al*, 1999), breast (Jager *et al*, 1999) and prostate cancer, and T cell leukemia (Itoh *et al*, 1999). No evidence for the implication of the genes in cancer or autoimmune states has been reported for the remaining antigens.

One possible reason for the immunogenicity of self-proteins is structural changes, resulting from mutations, translocations or the experiment of splice variants. Although we identified several sequence variations in three tumour-derived cDNAs (NUCB2, endomucin-2 and CGI-96), analysis of these sequences in tumour and normal tissue specimens indicates that these variations represent allelic polymorphisms and are not likely to be associated with the immunogenicity of these proteins. Nevertheless, three-nucleotide deletion within the coding region of NUCB2 that we detected with very high frequency among the cancer patients, is very interesting finding. As a result of the deletion Gln is omitted from the hydrophobic C-terminus of the protein. Moreover, comparison of mRNA levels between cancerous and adjacent non-cancerous tissues by RT–PCR showed that the expression of NUCB2 is downregulated in 50% of gastric tumours. NUCB2 is a Ca^2+^ binding protein, which also contains putative DNA binding domain, two helix–loop–helix motifs, EF-hands and leucine zipper (Barnikol-Watanabe *et al*, 1994; Kroll *et al*, 1999). It interacts with the postmitotic growth suppressor necdin and is proposed to be involved in the regulation of survival and death of postmitotic cells by controlling Ca^2+^ homeostasis in the cytoplasm (Taniguchi *et al*, 2000). Taken together, NUCB2 shows some characteristics of tumour suppressor gene but further studies investigating the functional significance of the 3-nucleotide deletion and downregulation of NUCB2 expression are required.

Alterations in the pattern and efficiency of alternative splicing of several pre-mRNAs (e.g. CD44, BRCA1, WT-1) have been implicated in tumorigenesis and correlate with tumour progression (Cooper and Dougherty, 1995; Liu *et al*, 2001). De-regulation of splice site selection may serve as an additional mechanism for the generation of protein diversity contributing to the selection of more aggressive tumour cells (Cooper and Mattox, 1997; Philips and Cooper, 2000). We identified several previously unknown splice variants of two genes – TACC1 represented by clone Ga55, and uncharacterised gene HSPC232 represented by clone Ga50. TACC1 is a recently identified gene, a member of TACC protein family that is involved in the regulation of interaction between centrosomes and microtubules (Gergely *et al*, 2000a). Overexpression of TACC1 in mouse fibroblasts results in cellular transformation and anchorage independent growth (Still *et al*, 1999). In *Drosophila* embryos, overexpression of TACC domain led to accumulation of microtubule asters and a complete failure in development of the embryos (Lee *et al*, 2001). In contrast, embryos carrying a mutation in *d-tacc* gene that decrease the level of D-TACC protein have shorter centrosomal microtubules and they also develop severe mitotic defects (Gergely *et al*, 2000b). These data show that perturbation of TACC gene expression may provoke various effects ranging from a genetic instability and missegregation of chromosomes to cell death. In the current study we identified a novel TACC1 isoform generated by inclusion of an alternative 36 bp exon. This isoform was strongly expressed in three of 20 specimens of gastric cancer tissues while it was undetectable in the adjacent gastric epithelium and normal stomach epithelium. From normal adult tissues it was predominately expressed in brain. We propose that alteration in the pattern of TACC1 mRNA splicing in cancer cells might lead to perturbation of TACC1 function. Whether the ectopic expression of this isoform served as a stimulus for antibody production in the patient also remains to be established. Interestingly, TACC1 and TACC2 have also have been detected by SEREX in gastric cancer by Yuichi Obata (SEREX database).

The largest category of SEREX antigens occur as overexpressed gene products. Gene amplification, elevated transcription and stability of mRNA or protein may lead to accumulation of protein and induction of immune response by exceeding the number of triggered TCRs required for T cell activation (Viola and Lanzavecchia, 1996; Lanzavecchia *et al*, 1999). In order to search for overexpressed antigens, we used semi-quantitative RT–PCR to examine the mRNA levels of the identified antigens in gastric cancer and adjacent non-cancerous tissues. Relative overexpression of Ga27 (granulin), Zg15 (orthologous of dog's C3VS gene) and Ga19 (orthologous to mouse Tbdn-1 gene) in gastric cancer was observed. Upregulation of the growth factor granulin has been shown in brain tumours (Liau *et al*, 2000) and in highly tumourigenic cell lines (Zhang and Serrero, 1998). Granulin has been shown to have mitogenic activity in fibroblasts, epithelial cells and glioblastoma cell lines (Xu *et al*, 1998; Liau *et al*, 2000; Lu and Serrero, 2000). Zg15 represents a human orthologue of dog's C3VS gene, its function is unknown but it is upregulated by mitogenic stimulation in thyroid cells (Wilkin *et al*, 1996). Thus, increased proliferation of tumour cells alone could account for the overexpression of Zg15 in cancerous tissues. In theory, it could be considered as a proliferation marker and its prognostic significance remains to be determined. Another gene that we found to be upregulated in three of 20 gastric cancer specimens is human orthologue of the mouse tbdn-1 gene encoding putative acetyltransferase. The levels and tissue distribution of tbdn-1 suggest that it may be involved in regulation of vascular and hematopoietic development and angiogenesis. Tbdn-1 is expressed at high levels at day 8 of gestation, when yolk sac vasculogenesis and blood island formation is peaking but downregulated at day 10 when formation of larger coalesced vitelline vessels occurs. In adult mouse it is expressed in atrial endocardium, bone morrow and ovarian follicles only (Gendron *et al*, 2000). Although we observed relatively high expression of Tbdn-1 in spleen, colon, stomach, testis and ovary, it could be of interest to investigate which cell populations within a tumour overexpress it and whether this acetyltransferase has a role in tumour neovascularisation

In conclusion, a number of the antigens that we have identified in this study may have a functional role in cancer aetiology or progression, and the altered expression pattern of these genes may provide the immunogenic stimuli in these patients. However other possibilities, for example, unidentified mutations or altered sub-cellular localisation which have been recently shown for β-actin in apoptotic breast cancer cells (Hansen *et al*, 2001), cannot be excluded. Further studies will be focused on exploration of the possible functional differences between TACC1 isoforms, definition of cell populations that express Tbdn-1 acetyltransferase and investigation of its possible role in tumour angiogenesis. It will also be of interest to elucidate the possible role of NUCB2 in tumour progression and the functional significance of the peculiar three-nucleotide polymorphysm. In addition, serological responses to seven antigens (including TACC1, NUCB2, Tbdn-1 and granulin etc.) were restricted to cancer patients and investigation of their relevance for sero-diagnosis or prognosis is in progress.

## References

[bib1] Barnikol-WatanabeSGrossNAGotzHHenkelTKarabinosAKratzinHBarnikolHUHilschmannN1994 Human protein NEFA, a novel DNA binding/EF-hand/leucine zipper protein. Molecular cloning and sequence analysis of the cDNA, isolation and characterization of the protein Biol Chem Hoppe Seyler 375 497512 7811391 10.1515/bchm3.1994.375.8.497

[bib2] BolivarJGuelmanSIglesiasCOrtizMValdiviaMM1998 The fragile-X-related gene FXR1 is a human autoantigen processed during apoptosis J Biol Chem 273 1712217127 9642279 10.1074/jbc.273.27.17122

[bib3] BrassNHeckelDSahinUPfreundschuhMSybrechtGWMeeseE1997 Translation initiation factor eIF-4gamma is encoded by an amplified gene and induces an immune response in squamous cell lung carcinoma Hum Mol Genet 6 3339 9002667 10.1093/hmg/6.1.33

[bib4] CooperDLDoughertyGJ1995 To metastasize or not? Selection of CD44 splice sites Nat Med 1 635637 7585142 10.1038/nm0795-635

[bib5] CooperTAMattoxW1997 The regulation of splice-site selection, and its role in human disease Am J Hum Genet 61 259266 9311728 10.1086/514856 PMC1715899

[bib6] GendronRLAdamsLCParadisH2000 Tubedown-1, a novel acetyltransferase associated with blood vessel development Dev Dyn 218 300315 10842358 10.1002/(SICI)1097-0177(200006)218:2<300::AID-DVDY5>3.0.CO;2-K

[bib7] GergelyFKarlssonCStillICowellJKilmartinJRaffJW2000a The TACC domain identifies a family of centrosomal proteins that can interact with microtubules Proc Natl Acad Sci USA 97 1435214357 11121038 10.1073/pnas.97.26.14352 PMC18922

[bib8] GergelyFKiddDJeffersKWakefieldJGRaffJW2000b D-TACC: a novel centrosomal protein required for normal spindle function in the early Drosophila embryo EMBO J 19 241252 10637228 10.1093/emboj/19.2.241 PMC305558

[bib9] HanahanDWeinbergRA2000 The hallmarks of cancer Cell 100 5770 10647931 10.1016/s0092-8674(00)81683-9

[bib10] HansenMHNielsenHDitzelHJ2001 The tumor-infiltrating B cell response in medullary breast cancer is oligoclonal and directed against the autoantigen actin exposed on the surface of apoptotic cancer cells Proc Natl Acad Sci USA 98 1265912664 11606714 10.1073/pnas.171460798 PMC60110

[bib11] HirakataMSuwaANagaiSKronMATrieuEPMimoriTAkizukiMTargoffIN1999 Anti-KS: identification of autoantibodies to asparaginyl-transfer RNA synthetase associated with interstitial lung disease J Immunol 162 23152320 9973509

[bib12] ItohMWatanabeMYamadaYFurukawaKTaniguchiMHataTSchmittMIkedaHYamaguchiMOhnoTNakashimaKShikuH1999 HUB1 is an autoantigen frequently eliciting humoral immune response in patients with adult T cell leukemia Int J Oncol 14 703708 10087317 10.3892/ijo.14.4.703

[bib13] JagerDStockertEJagerEGureAOScanlanMJKnuthAOldLJChenYT2000a Serological cloning of a melanocyte rab guanosine 5′-triphosphate- binding protein and a chromosome condensation protein from a melanoma complementary DNA library Cancer Res 60 35843591 10910072

[bib14] JagerDStockertEScanlanMJGureAOJagerEKnuthAOldLJChenYT1999 Cancer-testis antigens and ING1 tumor suppressor gene product are breast cancer antigens: characterization of tissue-specific ING1 transcripts and a homologue gene Cancer Res 59 61976204 10626813

[bib15] JagerEGnjaticSNagataYStockertEJagerDKarbachJNeumannARieckenbergJChenYTRitterGHoffmanEArandMOldLJKnuthA2000b Induction of primary NY-ESO-1 immunity: CD8+ T lymphocyte and antibody responses in peptide-vaccinated patients with NY-ESO-1+ cancers Proc Natl Acad Sci USA 97 1219812203 11027314 10.1073/pnas.220413497 PMC17318

[bib16] JagerENagataYGnjaticSWadaHStockertEKarbachJDunbarPRLeeSYJungbluthAJagerDArandMRitterGCerundoloVDupontBChenYTOldLJKnuthA2000c Monitoring CD8 T cell responses to NY-ESO-1: correlation of humoral and cellular immune responses Proc Natl Acad Sci USA 97 47604765 10781081 10.1073/pnas.97.9.4760 PMC18306

[bib17] KrollKAOtteSHirschfeldGBarnikol-WatanabeSGotzHSternbachHKratzinHDBarnikolHUHilschmannN1999 Heterologous overexpression of human NEFA and studies on the two EF- hand calcium-binding sites Biochem Biophys Res Commun 260 18 10381334 10.1006/bbrc.1999.0867

[bib18] LanzavecchiaALezziGViolaA1999 From TCR engagement to T cell activation: a kinetic view of T cell behavior Cell 96 14 9989490 10.1016/s0092-8674(00)80952-6

[bib19] LeeMJGergelyFJeffersKPeak-ChewSYRaffJW2001 Msps/XMAP215 interacts with the centrosomal protein D-TACC to regulate microtubule behaviour Nat Cell Biol 3 643649 11433296 10.1038/35083033

[bib20] LiauLMLalloneRLSeitzRSBuznikovAGreggJPKornblumHINelsonSFBronsteinJM2000 Identification of a human glioma-associated growth factor gene, granulin, using differential immuno-absorption Cancer Res 60 13531360 10728698

[bib21] LiuHXCartegniLZhangMQKrainerAR2001 A mechanism for exon skipping caused by nonsense or missense mutations in BRCA1 and other genes Nat Genet 27 5558 11137998 10.1038/83762

[bib22] LuRSerreroG2000 Inhibition of PC cell-derived growth factor (PCDGF, epithelin/granulin precursor) expression by antisense PCDGF cDNA transfection inhibits tumorigenicity of the human breast carcinoma cell line MDA-MB-468 Proc Natl Acad Sci USA 97 39933998 10760271 10.1073/pnas.97.8.3993 PMC18130

[bib23] PfreundschuhM2000 Exploitation of the B cell repertoire for the identification of human tumor antigens Cancer Chemother Pharmacol 46SupplS3S7 10950139 10.1007/pl00014046

[bib24] PhilipsAVCooperTA2000 RNA processing and human disease Cell Mol Life Sci 57 235249 10766020 10.1007/PL00000687 PMC11147109

[bib25] SahinULiGTureciOPfreundschuhM2001 Recognition of human tumors: SEREX expression cloning to identify tumour antigensInCancer ImmunologyRobins RA, Rees RC (eds)pp4557Dordrecht/Boston/London: Kluwer Academic Publishers

[bib26] SahinUTureciOSchmittHCochloviusBJohannesTSchmitsRStennerFLuoGSchobertIPfreundschuhM1995 Human neoplasms elicit multiple specific immune responses in the autologous host Proc Natl Acad Sci USA 92 1181011813 8524854 10.1073/pnas.92.25.11810 PMC40492

[bib27] ScanlanMJChenYTWilliamsonBGureAOStockertEGordanJDTureciOSahinUPfreundschuhMOldLJ1998 Characterization of human colon cancer antigens recognized by autologous antibodies Int J Cancer 76 652658 9610721 10.1002/(sici)1097-0215(19980529)76:5<652::aid-ijc7>3.0.co;2-p

[bib28] ScanlanMJGordanJDWilliamsonBStockertEBanderNHJongeneelVGureAOJagerDJagerEKnuthAChenYTOldLJ1999 Antigens recognized by autologous antibody in patients with renal-cell carcinoma Int J Cancer 83 456464 10508479 10.1002/(sici)1097-0215(19991112)83:4<456::aid-ijc4>3.0.co;2-5

[bib29] StillIHHamiltonMVincePWolfmanACowellJK1999 Cloning of TACC1, an embryonically expressed, potentially transforming coiled coil containing gene, from the 8p11 breast cancer amplicon Oncogene 18 40324038 10435627 10.1038/sj.onc.1202801

[bib30] TaniguchiNTaniuraHNiinobeMTakayamaCTominaga-YoshinoKOguraAYoshikawaK2000 The postmitotic growth suppressor necdin interacts with a calcium- binding protein (NEFA) in neuronal cytoplasm J Biol Chem 275 3167431681 10915798 10.1074/jbc.M005103200

[bib31] ThellinOZorziWLakayeBDe BormanBCoumansBHennenGGrisarTIgoutAHeinenE1999 Housekeeping genes as internal standards: use and limits J Biotechnol 75 291295 10617337 10.1016/s0168-1656(99)00163-7

[bib32] TohYOkiEOdaSTokunagaEOhnoSMaeharaYNicolsonGLSugimachiK1997 Overexpression of the MTA1 gene in gastrointestinal carcinomas: correlation with invasion and metastasis Int J Cancer 74 459463 9291440 10.1002/(sici)1097-0215(19970822)74:4<459::aid-ijc18>3.0.co;2-4

[bib33] TureciOSahinUPfreundschuhM1997 Serological analysis of human tumor antigens: molecular definition and implications Mol Med Today 3 342349 9269687 10.1016/s1357-4310(97)01081-2

[bib34] ViolaALanzavecchiaA1996 T cell activation determined by T cell receptor number and tunable thresholds Science 273 104106 8658175 10.1126/science.273.5271.104

[bib35] WeisingerGGavishMMazurikaCZinderO1999 Transcription of actin, cyclophilin and glyceraldehyde phosphate dehydrogenase genes: tissue- and treatment-specifity Biochim Biophys Acta 1446 225232 10524197 10.1016/s0167-4781(99)00091-3

[bib36] WilkinFSavonetVRadulescuAPetermansJDumontJEMaenhautC1996 Identification and characterization of novel genes modulated in the thyroid of dogs treated with methimazole and propylthiouracil J Biol Chem 271 2845128457 8910471 10.1074/jbc.271.45.28451

[bib37] XuSQTangDChamberlainSPronkGMasiarzFRKaurSPriscoMZanocco-MaraniTBasergaR1998 The granulin/epithelin precursor abrogates the requirement for the insulin-like growth factor 1 receptor for growth in vitro J Biol Chem 273 2007820083 9685348 10.1074/jbc.273.32.20078

[bib38] ZhangHSerreroG1998 Inhibition of tumorigenicity of the teratoma PC cell line by transfection with antisense cDNA for PC cell-derived growth factor (PCDGF, epithelin/granulin precursor) Proc Natl Acad Sci USA 95 1420214207 9826678 10.1073/pnas.95.24.14202 PMC24351

[bib39] ZhongHSimonsJW1999 Direct comparison of GAPDH, beta-actin, cyclophilin, and 28S rRNA as internal standards for quantifying RNA levels under hypoxia Biochem Biophys Res Commun 259 523526 10364451 10.1006/bbrc.1999.0815

